# Comment on ‘Impact of sarcopenia on outcomes in surgical patients: a systematic review and meta-analysis’

**DOI:** 10.1097/JS9.0000000000000846

**Published:** 2023-10-26

**Authors:** Xiao-Ming Zhang

**Affiliations:** Department of Emergency, The People’s Hospital of Baoan Shenzhen, Shenzhen, People’s Republic of China

*Dear Editor*,

Recently, we read a systematic review and meta-analysis, with great interest, by Knoedler and his colleagues^1^, who explored the association between sarcopenia and outcomes among surgical patients. The results indicated that surgical patients with sarcopenia had a significantly higher risk of adverse outcomes, including mortality, complications, and prolonged hospital stays. This underscores the importance of assessing and managing sarcopenia during the perioperative period. This study exhibited several strengths, including the largest sample size to date. Additionally, the study adhered to the PRISMA (Preferred Reporting Items for Systematic Reviews and Meta-Analyses) guidelines in its methodology, and the statistical analyses included subgroup analysis, meta-regression, and an assessment of publication bias, making it comprehensive in its approach. This finding can assist healthcare providers in establishing comprehensive programs to make informed decisions when performing surgical operations. The knowledge has the potential to enhance surgical patient outcomes and optimize resource allocation within the healthcare system. However, certain underlying concerns necessitate further clarification.

Firstly, the authors combined the association between sarcopenia and adverse outcomes such as mortality, complications, and discharge to home. The results indicated that sarcopenia increased the risk of mortality and complications while decreasing the likelihood of home discharge compared to those without sarcopenia. However, caution is needed when interpreting these findings because these effect sizes were crude and did not truly reflect the independent associations, which could overestimate the effect size for these associations. As we know, establishing the independent association between sarcopenia and mortality requires adjusting for potentially confounding factors related to both sarcopenia and mortality. Typically, original studies present this independent association using multivariate logistic regression. Therefore, the authors could extract the independent effect sizes [OR (odds ratio), RR (relative risk), and HR (hazard ratio)] from these original studies and pooled them to genuinely reflect the association between sarcopenia and adverse outcomes, particularly when the study design is observational and cannot eliminate potential confounding factors. In Knoedler’s study, they might have explicitly stated that they calculated crude effect sizes in the methods.

Secondly, based on the extensive sample size and multiple studies, the authors could have addressed an important question: what is the prevalence of sarcopenia among surgical patients? By pooling raw data from each original study, the results showed that the pooled prevalence of sarcopenia among surgical patients was 37% [95% confidence interval (95% CI): 35–39%], showed in Table 1, which was higher than that among community-dwelling people^2^ (12.9%, 95% CI: 9.9–15.9%). The prevalence was similar among patients with cardiovascular diseases, with a prevalence of 35% (95% CI: 28–42%)^3^. Furthermore, conducting subgroup analyses based on the assessment methods for the prevalence of sarcopenia in surgical patients can provide important information. The results indicated that the highest prevalence of sarcopenia was 54% (95% CI: 33–74%, *n*=3) when Psoas Muscle Mass was used to assess low muscle mass, followed by Skeletal Muscle Mass (41%, 95% CI: 37–46%, *n*=4), Psoas Muscle Index (40%, 95% CI: 35–45%, *n*=46), Skeletal Muscle Index (39%, 95% CI: 36–42%, *n*=151), Psoas Muscle Area (37%, 95% CI: 32–43%, *n*=24), Total Psoas Volume (36%, 95% CI: 15–60%, *n*=2), Skeletal Muscle Area (35%, 95% CI: 28–42%, *n*=25), Masseter Muscle Area (34%, 95% CI: 7–68%, *n*=2), Total Psoas Index (29%, 95% CI: 25–32%, *n*=10), and Total Psoas Area (27%, 95% CI: 23–31%, *n*=23), showed in Table 1. This finding suggests that the prevalence of sarcopenia varies depending on the assessment method used. Future clinical guidelines should be established by authoritative and experienced experts to recommend the appropriate tools for confirming low muscle mass, taking into consideration the clinical scenario.

**Table 1:** The pooled prevalence of sarcopenia among surgical patients and subgroup analysis based on assessment methods.

	Number of studies	Pooled (%)	95% CI (%)	*P*	*I*^2^ (%)
Overall prevalence	295	37	35–39	<0.01	97
Subgroup analysis based on assessment methods					
TPA	23	27	23–31	<0.01	84
SMI	151	39	36–42	<0.001	97
PMI	46	40	35–45	<0.01	95
PMA	24	37	32–43	<0.01	97
SMA	25	35	28–42	<0.01	96
TPV	2	36	15–60	<0.01	96
SMM	4	41	37–46	0.15	44
PAI	1	30	25–36	–	–
TPI	10	29	25–32	<0.01	74
PMM	3	54	33–74	<0.01	95
PeMI	2	24	16–33	<0.01	85
TMT	1	56	47–64	–	–
MMA	2	34	7–68	<0.01	95

MMA, Masseter Muscle Area; PeMI, Pectoralis Muscle Index; PMA, Psoas Muscle Area; PMI, Psoas Muscle Index; PMM, Psoas Muscle Mass; SMA, Skeletal Muscle Area; SMI, Skeletal Muscle Index; SMM, Skeletal Muscle Mass; TMT, Temporal Muscle Thickness; TPA, Total Psoas Area; TPI, Total Psoas Index; TPV, Total Psoas Volume.

Thirdly, we suggest that the authors provide detailed information about the cutoff points used to define sarcopenia in each original study. Different definitions of sarcopenia, such as AWGS (Asian Working Group for Sarcopenia), EWGSOP (European Working Group on Sarcopenia in Older People), and FNIH (Foundation for the National Institutes of Health), have varying cutoff points for confirming low muscle mass^3^. While most original studies used computed tomography to assess muscle mass, they employed various assessment methods such as SMI (Skeletal Muscle Index), TPA (Total Psoas Area), PMI (Psoas Muscle Index), SMA (Skeletal Muscle Area), TPI (Total Psoas Index), PMM (Psoas Muscle Mass), SMM (Skeletal Muscle Mass). Therefore, we strongly advise the authors to conduct subgroup analyses for adverse outcomes based on the assessment methods to determine which method is the most sensitive for assessing the impact of sarcopenia on adverse outcomes among surgical patients.

Lastly, the authors acknowledged that one of the limitations of this systematic review and meta-analysis was that most of the studies were retrospective and single-center. Therefore, the authors could have performed a subgroup analysis based on the type of center (single-center vs. multiple-center) to identify whether there was a significant difference in this variable.

## Ethical approval

Not applicable.

## Consent

Not applicable.

## Sources of funding

No funding.

## Author contribution

X.-M.Z. is responsible for study design and writing.

## Conflicts of interest disclosure

There are no conflicts of interest.

## Research registration unique identifying number (UIN)

Not applicable.

## Guarantor

Xiao-Ming Zhang is the guarantor.

## Data availability statement

The data in this study can be obtained from the public internet.

## Provenance and peer review

Commentary, internally reviewed.
